# Fifteen years of the cologne medical model study course: has the expectation of increasing student interest in general practice specialization been fulfilled?

**DOI:** 10.3205/zma001266

**Published:** 2019-10-15

**Authors:** Heike Zims, Yassin Karay, Peter Neugebauer, Stefan Herzig, Christoph Stosch

**Affiliations:** 1 University of Cologne, Faculty of Medicine, Office of the Dean of Studies, Cologne, Germany; 2 Technische Hochschule Cologne, Cologne, Germany

**Keywords:** model study course, general practice, GP shortage, Medical Licensure Act, standard degree course

## Abstract

**Background: **The 2002 Medical Licensure Act gave German universities certain freedoms for reforming their medical degree courses. The Medical Faculty of the University of Cologne took advantage of this opportunity and introduced a model study course in the winter semester 2003/04 through §41 of the Medical Licensure Act. One of the main reasons for this was that back then there was an increasing shortage of doctors in clinical curative medicine and GP primary care. This study investigates whether the introduction of the Cologne Model Study Course (MSG) can show stronger interest in curative medical work (especially General Practice) compared to students of the standard degree course (RSG).

**Methodology: **The proof of added value was examined through graduate surveys conducted at the University of Cologne and through the proportion of students who completed the PY elective rotation “General Practice”. The students of the standard degree course (start of studies prior to winter semester 2003/2004) were compared with students of the model study course (start of studies from winter semester 2003/04 onwards). Measurements were carried out using descriptive frequency tables and correlation analyzes according to Spearman.

**Results: **The students’ interest in curative medicine was already high (91%) even before the model study course was introduced and increased only slightly (to 91.9%). There is also only a slight increase in specialization in General Practice (RSG=5.9% vs. MSG=9.2%). However, selection of rotations in General Practice was significantly increased (RSG=1.9% vs. MSG=3.4%, r=0.046 **, p<0.005).

**Conclusion:** The Cologne Model Study Course in Human Medicine has increased awareness of the subject of General Practice among students through a large number of curricular changes. The fact that only marginal effects can be demonstrated shows once more the strong dependence of choosing General Medicine as a career path on other factors (such as gender or the presence of positive role models) and emphasizes the necessity of promoting General Practice student education not only through increased curricular mapping but by additional innovative concepts to maximize the status of General Practice from the perspective of students.

## Zusammenfassung

**Hintergrund:** Die Approbationsordnung für Ärzte von 2002 eröffnete den deutschen Universitäten diverse Spielräume, ihre Medizinstudiengänge zu reformieren. Die Medizinische Fakultät der Universität zu Köln nutzte diese Möglichkeit und führte im Wintersemester 2003/04 mit Hilfe des §41 ÄAppO einen Modellstudiengang ein. Einer der Hauptgründe dafür war der seinerzeit zunehmende Ärztemangel in der klinisch-kurativen Medizin und der hausärztlichen Grundversorgung. Im Rahmen dieser Studie wird untersucht, ob durch die Einführung des Kölner Modellstudiengangs (MSG) eine stärkere Hinwendung der Studierenden zu kurativ-medizinischen Arbeitsbereichen (insbesondere Allgemeinmedizin) im Vergleich zu Studierenden des Regelstudiengangs (RSG) nachgewiesen werden kann.

**Methodik: **Der Mehrwertnachweis wurde mit Hilfe der an der Universität zu Köln durchgeführten Absolventenbefragungen und mit Hilfe des Anteils der absolvierten PJ-Wahltertiale „Allgemeinmedizin“ untersucht. Verglichen wurden die Studierenden des Regelstudiengangs (Studienbeginn vor Wintersemester 2003/2004) mit Studierenden des Modellstudiengangs (Studienbeginn ab Wintersemester 2003/04). Die Messung erfolgte mit deskriptiven Häufigkeitstabellen sowie Korrelationsanalysen nach Spearman.

**Ergebnisse:** Die Hinwendung der Studierenden zur kurativen Medizin lag insgesamt schon vor dem Modellstudiengang auf einem hohen Niveau (91%) und konnte nur gering gesteigert werden (auf 91,9%). Auch bei der Weiterbildung im Fach Allgemeinmedizin ist nur eine leichte Steigerung nachzuweisen (RSG=5,9% vs. MSG=9,2%). Die Wahl der Tertiale in der Allgemeinmedizin konnte allerdings signifikant gesteigert werden (RSG=1,9% vs. MSG=3,4%; r=0,046**; p<0,005).

**Schlussfolgerung: **Der Kölner Modellstudiengang Humanmedizin hat mit einer Vielzahl von curricularen Änderungen das Fach Allgemeinmedizin stärker in den Fokus der Studierenden gerückt. Dass sich trotzdem nur marginale Effekte nachweisen lassen, zeigt einmal mehr die starke Abhängigkeit der Berufswahl „Allgemeinmedizin“ von weiteren Faktoren (beispielsweise dem Geschlecht oder dem Vorhandensein positiver Rollenmodelle) und betont die Notwendigkeit, die studentische Ausbildung in der Allgemeinmedizin nicht nur durch vermehrte curriculare Abbildung zu fördern, sondern durch weitere innovative Konzepte den Stellenwert der Allgemeinmedizin aus Sicht der Studierenden zu maximieren.

## 1. Introduction

The reform of medical studies at the Medical Faculty of the University of Cologne was of particular importance even before the introduction of the model study course. In the years before the new Medical Licensure Act came into force in 2002 [https://www.gesetze-im-internet.de/_appro_2002/BJNR240500002.html], initiatives to improve teaching were regularly implemented, such as the introduction of:

- Innovative examination courses
- Interdisciplinary PbL seminars
- Clinical block internships and block seminars
- Medical communication and skills training (in preparation for the future creation of a Skills Lab)
- Comprehensive introduction of block internships in a network of General Practice teaching practices

At that time the background to these reform measures was a recognition of a “discrepancy between study-related qualifications and required professional qualifications” among graduates [1] as well as an increasing shortage of doctors in clinically curative medicine and primary care [1], [2]. For this reason, a Delphi survey on the objectives for medical studies in Cologne and their medical career was carried out at the Cologne Medical Faculty among the lecturers and also students in the winter semester 2001/2002 [2].

**Figure 1:**
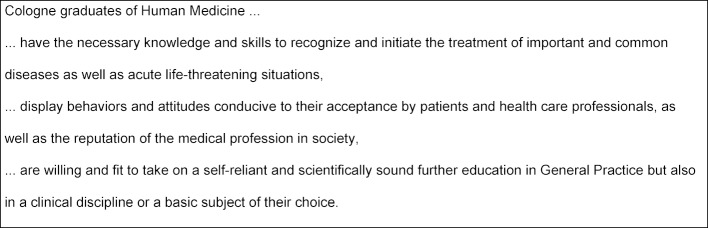
Mission Statement for Medical Education (Source: Study Regulations 2008 [3])

The data analysis of this multi-level, systematic survey enabled the development of a faculty-specific mission statement for Cologne graduates. This became the curricular basis for the development of the competence and outcome-oriented Cologne Model Study Course (see figure 1 (Fig. 1)):

The resulting faculty mission statement has to some extent already gone beyond the general goals of education demanded in the old licensing regulations [https://www.gesetze-im-internet.de/_appro_2002/BJNR240500002.html]. At the same time, many of the changes prescribed in the new Licensure Act (for example, the introduction of a block internship in General Practice, the strengthening of practical instruction with patients and the offer of elective courses) were already enshrined in the curriculum of the Cologne standard degree course [3]. So it was consistent at that time to implement further reform steps (for example, scientific projects, curricular integration of clinical content in the phase of studies - see below) by introducing a Cologne-specific model study course, which started in the winter semester 2003/2004.

On the basis of the Cologne mission statement for teaching, the content and structural framework of the model study course was developed in the relevant commissions, which are composed in equal parts of students, academic staff and professors. For the development of the curriculum the guiding principle was that the new innovative teaching elements had to be linked with the existing resources and the content strengths of medical studies in Cologne in a cost-effective manner, also with a view to an increase in General Practice competencies. As special features and in contrast to other model study courses

1. the traditional, discipline-based range of subjects as the core curriculum had its number of hours reduced and was supplemented with patient-related, interdisciplinary teaching of the most important symptoms and reasons for appointments in the interdisciplinary so-called “competence areas” and “compulsory elective blocks” (for explanation see below),
2. the longitudinal training in practical skills was integrated into the curriculum and professionalized through the establishment of the “Cologne Interprofessional Skills Lab and Simulation Center (KISS)”, one of the first Skills Labs in the German-speaking world; and
3. two compulsory scientific projects were introduced to encourage students towards more in-depth scientific work and understanding.

In addition to improved professional skills and increased scientific output, the Cologne Model Study Course aims are both to promote an increased focus on curative medical career paths with a special emphasis on General Practice. Starting with a description of the elements of the model study course and using a retrospective cross-sectional analysis contrasting students of the Cologne standard and model study course, this article examines if the presented measures succeeded in encouraging students of the model study course towards curative medical career paths. Because of the current health policy discourse (for example the Masterplan for Medical Studies 2020, proportion of rural doctors, introduction of M3 exam in General Practice [4]), the focus is in particular on the “promotion of General Practice”.

For example, the German council of experts for the assessment of developments in the health care sector predicts a shortfall of up to 18,000 GPs in Germany over the next few years in view of the low number of young graduates in General Practice (with at the same time an increasing number of graduates) and the increasing number of currently working GPs retiring [5].

## 2. Materials and Methods

### 2.1. Structure of the model study course

The study of medicine in Germany, including exam preparation, takes twelve semesters plus three months [6]. In contrast to other model study courses, the Cologne Model Study Course consists of a first (four semester) and a second (six semester) study phase and the subsequent Practical Year (two semesters) where clinical knowledge was integrated (N Model) in particular into the first study phase (competence areas, skills training and “StudiPat”, see below). In terms of the curriculum, the Cologne model could be described as a combined H & N model, since in addition to integration there is also a summative interim examination (Basic Medical Examination, H model) (on “N vs. H model”, see [7]). 

The curricular implementation of the mission statement for teaching in the Cologne model course is based on four pillars, of which only the first pillar was systematically anchored in the previous curriculum:

#### 2.1.1. Subject-related teaching of classical disciplines

The basic structure of the curriculum is similar to the standard degree courses as regards the classical disciplines, cross-sectional areas and block internships which in the second phase of studies are systematically implemented as block teaching: The theoretical basics are taught in relation to content in the first half of the semester, while the second half features practical training with patient contact.

Competences are verified through grading of various written, oral and practical exams and for the block internships, following the Medical Licensure Act, through OSCE exams (Objective Structured Clinical Examination) or a log book exam in General Practice.

##### 2.1.2. Interdisciplinary teaching in “competence areas”

The above-mentioned discipline-based curriculum is supplemented by consistently interdisciplinary teaching units in the so-called “competence areas” (KF), which represent an essential innovation in the Cologne Model Study Course. In general the most important diagnoses, symptoms and reasons for appointments in General Practice are taught in an interdisciplinary way using different teaching formats (lectures, seminars, internships) in 60 compact courses (five to ten teaching hours each), from the first to the tenth semester. The content of the competence areas creates a continuous focus on patients in General Practice and are intended to prepare students for the needs of “medical practice” and to promote an interdisciplinary understanding.

The “competence areas” were developed by means of “curriculum mapping” [1]. The teaching content of the previous compulsory curriculum was coded according to ICD-10 and a symptom key similar to the Dutch Blueprint [8] and then sorted and summarized using apparent redundancies (for the first phase of studies see table 1 (Tab. 1), for the second see attachment 1 ).

In the first four semesters, 18 competence areas integrate clinical aspects into the first phase of studies and correlate with teaching content in the first phase of studies. Formally, these replaced the bridge courses of the Medical Licensure Act “Seminars as integrated events” and “Seminars with clinical relevance”. For example, the competence area “Back pain” tackles topics in orthopedics, pharmacology, pain medicine and General Practice in relation to the basics in anatomy taught in the dissection course. The competence area “Vaccinations” builds on the content of the module “Virology” from practical biology course in the first phase of studies and combines this with the basics of prevention from the point of view of pediatrics and General Practice as well as the administration of intra-muscular injections.

In the second phase of studies, three competence areas complement the 14 cross-sectional areas (=42 competence areas, see attachment 1 ) according to the Medical Licensure Act, so that central topics of the respective areas are taught in an interdisciplinary manner [3]. 

To ensure successful learning, the competence areas are largely assessed through digital written supervised exams. 

Alternative types of exams can be found both in the first phase of studies in the forms of presentations and homework as well as in the second phase of studies as simulated exams (for example cardiopulmonary resuscitation) or homework. 

##### 2.1.3. Practice-oriented studies, learning practical skills

In addition to the bedside teaching prescribed in the Medical Licensure Act, the practical training of students in Cologne is characterized by a longitudinal education in primary medical care starting in the first semester and interpersonal skills in the “Cologne Interprofessional Skills Lab and Simulation Center (KISS)”. Skills training is an obligatory part of the curriculum. 

Based on the requirements in the work-oriented training phases (readiness for clinical traineeship in semesters 1-5 and PY in semesters 6-10), the students are also trained in clinical skills, emergency and patient consultation competences with the help of patient actors in simulation rooms that have been set up for this purpose. Numerous practical, especially formative feedback exams accompanying the course and summative final exams (simulation exams, OSCEs), spread over the entire course of studies, provide the students with feedback on their patient-related competences.

A special unit of the Cologne Model Study Course is the so-called study accompanying patient care (StudiPat). Starting from the first semester, all students receive the earliest possible patient contact in one of more than 250 GP surgeries in Cologne or its environs. Students are informed about the concept and procedures in a General Practice introductory session. Over eight semesters, regular contact is made (at least once a semester, after free scheduling between the participants) with the assigned patient and GP. The contacts are regularly documented and reflected on by the students. The portfolio contains the epicrisis, self-written medical reports, dossiers on medication, etc. and is assessed and evaluated by the Emphasis on General Practice at the University Hospital of Cologne. The grading is included in the overall assessment of the practical block in General Practice. In StudiPat students not only get to know the special aspects of outpatient care provided by SHI-accredited doctors but also the long-standing, trusting relationship between a GP and the patient. Based on the experiences in StudiPat, experiences in General Practice are longitudinally supplemented by the thematic focus on the competence areas competence (see above) and the elective block in General Practice and can be deepened in compulsory elective blocks and scientific projects (see 2.1.4) and in the elective rotation in the Practical Year.

##### 2.1.4. The elective curriculum and scientific rigor in medical studies

In order to increase the students engagement with the main research areas of the Medical Faculty and to teach scientific competencies early on, the graded electives of the Medical Licensure Act to be completed in the standard degree course were replaced by two “Scientific Projects” in the Cologne Model Study Course. In the first and second phase of studies, all students must complete a graded scientific work in the form of literature work, a clinical-statistical study or an experimental study by writing a project report. They are free to choose a field of research and under which professors they wish to work. This bridge between teaching and research aims to motivate students to increased and higher-quality scientific work and increased awareness of their own professional interests. It is possible to progress a scientific project towards a doctorate.

Further courses assigned to the elective curriculum take place as elective blocks, usually in the last two weeks of the semester. Depending on their interests, students can choose from a range of five to ten-hour teaching events in various subject areas and topics, primarily from the portfolio of the specialist courses of the current semester. At least one elective block must be taken each semester. There is no separate exam. 

#### 2.2. Sampling and procedure of data collection

To study signs of a stronger move towards General Practice, the following student cohorts were compared: 

The retrospective results of students who began to study medicine before the winter semester 2003/2004 or whose state medical examinations took place at the latest in the summer semester 2009 were used as a comparison cohort for the standard degree course. All cohorts who began to study medicine from winter semester 2003/04 on or whose state medical exam took place after the summer semester 2009 are considered the comparison cohort for the model study course. In order to minimize the inevitable mixed cohorts with slow students in the standard degree course, the most recent cohorts of the model study course were used as much as possible.

The proof of added value of an increased focus on curative medical career paths was analyzed with the help of the graduate survey carried out at the University of Cologne. The graduate surveys of the University of Cologne have been carried out since 2009 in cooperation with the International Center for Higher Education Research in Kassel (INCHER-Kassel). The staff of the Professional Center of the University of Cologne contact the graduates by letter one to two years after graduation and invite them to take part in an anonymous graduate survey. Graduates have the opportunity to choose between an online and a paper-based survey. For the purposes of this study, the survey data of the 2008 graduates (first regular evaluation by the university) will be used for the regular degree cohorts (N_RSG_=167) and for the model degree cohorts (N_MSG_=186) from 2014 to 2016, by evaluation the answers about employment situation and specialization at the time of the survey. The survey data for the 2017 graduation year were not available at the time of this evaluation.

In addition the proportion of completed General Practice rotations at all academic teaching surgeries/hospitals was determined retrospectively for the period 2007 to July 2009 for the standard degree course and from 2015 to 2017 for the model study course based on the annual service financing accounts. In total in the periods 2007 up to and including July 2009, 1,698 rotations were completed in the academic teaching surgeries and 2,062 in the years 2015 to 2017.

#### 2.3. Statistical evaluation

Frequency tables and correlation analyzes according to Spearman are used to measure the cohorts of the standard and model study courses. P-values below 0.05 are considered statistically significant. The evaluation was carried out using the statistical software SPSS (SPSS 25, SPSS Inc. Chicago, IL, USA).

## 3. Results

The response rate for the graduate surveys for the standard degree course (graduation years 2008 and 2009) is on average 28% and 19% for the model study course (graduation years 2014 to 2016). 

The question about the employment situation shows that the majority of Cologne graduates currently work in a curative medical field as part of their specialization. The absolute and relative frequency of “Yes” answers were analyzed for the question: “I’m working in health care”. Two years after their state examination, on average 91% of standard degree course graduates surveyed and 91.9% of graduates of the model study course work in a patient-related specialist area (see table 2 (Tab. 2)). The calculated correlation with Spearman is not significant (r=0.016, p=0.758).

The comparison between the examination cohorts of the two study courses with regard to specialization in General Practice at the time of the survey (specifically: What specialization are you currently pursuing?) (see table 3 (Tab. 3) ) shows that students of the model study course have increasingly opted for specialization in General Practice (RSG=5.9% vs. MSG=9.2%) but the measured correlation according to Spearman is also not significant (r=0.063, p=0.252). 

However, looking at the completed PY elective rotations it can be shown that, compared to the previous standard degree course, students in the model study course are increasingly taking the elective rotation in General Practice (RSG=1.9% vs. MSG=3.4%) (see table 4 (Tab. 4)). The measured correlation according to Spearman is significant at the 0.01 level and measures a low correlation between degree program and completed rotation in General Practice (r=0.046, p<0.01).

## 4. Discussion

With the introduction of the model study course in the winter semester 2003/04, the Medical Faculty of the University of Cologne set itself the goal of realizing the educational goals described in the mission statement [3]. Although the model study course pursues additional goals (scientific competence, interprofessionality, etc.), the main focus of this article is analyzing changes in the students’ interest in curative medicine and especially General Practice which the curricular changes introduced in the model study course aim to improve. 

Maintaining the systematic, subject-related teaching of classical disciplines described as a key feature of the Cologne Model Study Course offers students the opportunity to grasp important topics from across the tasks and care spectrum of the subject in question and to acquire scientific specialist communication skills. Also, the continuation of the traditional subject structure through the equivalence to standard degree courses ensures that the mobility of students (studying abroad, moving house) can be maintained largely without prolonging study time. 

As described by Woods et al. 2006 [9], focussing teaching content on specific symptoms and reasons for medical appointments in combination with teaching knowledge in basic subjects promotes differential diagnostic and therapeutic thinking, enabling students to make more precise diagnoses in clinical practice. In our opinion, the interdisciplinary focus of the competence areas offers the opportunity to perceive curative medicine from a patient-centered perspective in the form of General Practice and thus prepares students for the change of perspective during their Practical Year. This could be a reason why students increasingly turn to General Practice. 

The Masterplan for Medical Studies 2020 calls for an “early consistent patient orientation and the patient’s needs” [4], which is implemented in the Cologne model course through symptom-oriented knowledge transfer in the competence areas from the first semester on. The implementation of clinical topics within the scope of the competence areas in the first phase of studies enables a structured curricular implementation of the “seminars with clinical relevance” and “integrated seminars” called for in the Medical Licensure Act, with clear operationalized learning goals. A 2008 survey of 36 medical faculties in Germany revealed a very heterogeneous and in parts moderately structured implementation of called-for required seminars [10]. The nature of the implementation of clinical study content in the first phase of studies will therefore play an even greater role in the future because most of the teaching staff of pre-clinical subjects and subsequently also professors have no professional medical qualifications due to tariff differences between TV-L and TV-Ä.

With the introduction of competence areas, the teaching times for subject-specific lessons were further reduced in favor of skill training. However, the rough distribution of available teaching capacities between the individual subject areas is currently still very traditionally based on the previous ZVS specimen curriculum (ZVS=Central Student Clearing, now the Foundation for University Admissions), even if different hourly quotas are called for by students throughout Germany [11]. The “Reform of Competence Areas” carried out in 2013 shows that the process of curriculum development is continuously reviewed and adjusted if necessary. Following suggestions by the External Evaluation Committee, event criticism by students and focus group discussions in cooperation with the Curriculum Commission, the number of competence areas was significantly reduced (from 94 competence areas to 60) and the topics were oriented more consistently to symptoms and reasons for medical appointments. 

Compared to the previous standard degree course, the teaching of General Practice in the model course is much more extensive, introduced earlier and of higher quality. Nonetheless, data analysis on the increased uptake of General Practice only shows a positive trend with only partial significance, although the low response rate may have had led to distortions. 

It should be noted that significant changes in the Cologne Model Study Course have clear parallels to the Masterplan for Medical Studies 2020 [4]. Thus, the Cologne Medical Faculty already has a sufficient network of General Practice teaching surgeries in order to also offer comprehensive two-week block internships and PY elective rotations in General Practice in addition to the longitudinal anchoring of General Practice content in StudiPat. StudiPat, which by now is introduced in the first semester and runs over eight semesters, means that the Cologne Model Study Course already awards General Practice the “significance it is due in relation to its importance in health care” called for by the Masterplan for Medical Studies 2020 [4]. As described in several studies, early exposure to role models can be a key factor in the decision-making process of students regarding specialization [12], [13]. With the patient- and symptom-oriented teaching of the competence areas, a large part of the curriculum of medical studies in the first and second phases of study is aimed at outpatient care. The establishment of a Chair for General Practice in Cologne will lead to further important institutionalization of the subject in 2019, which should further increase the perceived value of the subject by the students [14]. 

It cannot be ruled out, however, that the described positive trend towards General Practice was not the result of the curricular measures described here but was rather influenced by stronger public awareness of the increasing shortage of GPs in Germany (history bias). A causal relationship between the Cologne Model Study Course and the trend towards General Practice cannot be proven.

It should therefore be questioned critically whether further expansion of compulsory teaching in the field of General Practice in future can really counteract the increasing shortage of GPs, as other factors have a significantly greater impact on choosing General Practice as a career path (especially in rural areas). For example, a study from Hanover in 2011 shows that the attitude to General Practice is more influenced by the characteristics of the students (mainly female students) than by the university curriculum (standard vs. model study course) [15]. It also remains doubtful to what extent universities and curricula can exert a fundamental influence on the graduates’ willingness to set up urgently needed GP practices [16], especially in rural areas. Press reports about rural doctors, who are faced with compensation claims in SHI care cases, for example because they have to do more home visits compared to urban GPs, are a strong deterrent even on students [17].

Since 2003 Cologne has already anticipated and implemented numerous requirements through the introduction of the model study course with regard to both the Masterplan for Medical Studies 2020 and the recommendations of the German Council for the Advancement of Medical Studies [18]. Other effects such as the teaching of scientific methods, the increased training of practical skills or subjectively perceived competence when entering the profession are currently the subject of further research. In addition, from a purely subjective point of view, the way in which the medical faculty has dealt with the model study course in Cologne must be awarded non-specific added value. Through the model study course, study and teaching have become institutionally more visible at the faculty (introduction of a curriculum commission, establishment of an internal and an external evaluation committee, staff development at the office of the Dean of Studies), so that the commitment to good teaching has become significantly more important to all concerned.

## Author contributions

H. Zims and Y. Karray share the first authorship. 

## Acknowledgements

This article on the Cologne Model Study Course in medicine is dedicated in grateful remembrance to the first Dean of Studies of the Medical Faculty of the University of Cologne, Univ. Prof. Dr. Jürgen Koebke. 

## Competing interests

The authors declare that they have no competing interests. 

## Supplementary Material

HOSPITAL Reform of competence areas in cross-sectional areas

## Figures and Tables

**Table 1:**
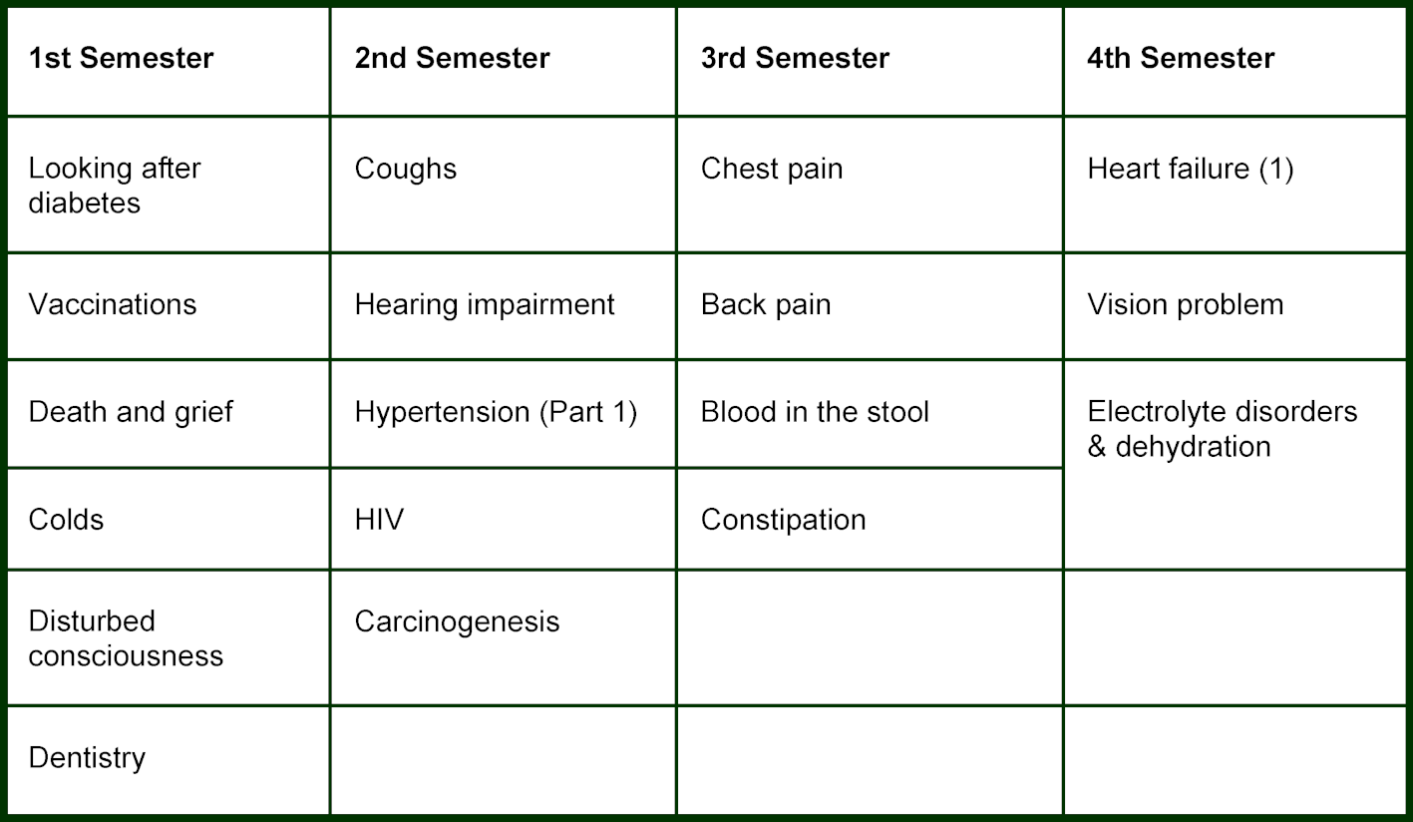
Competence areas in the first phase of studies

**Table 2:**
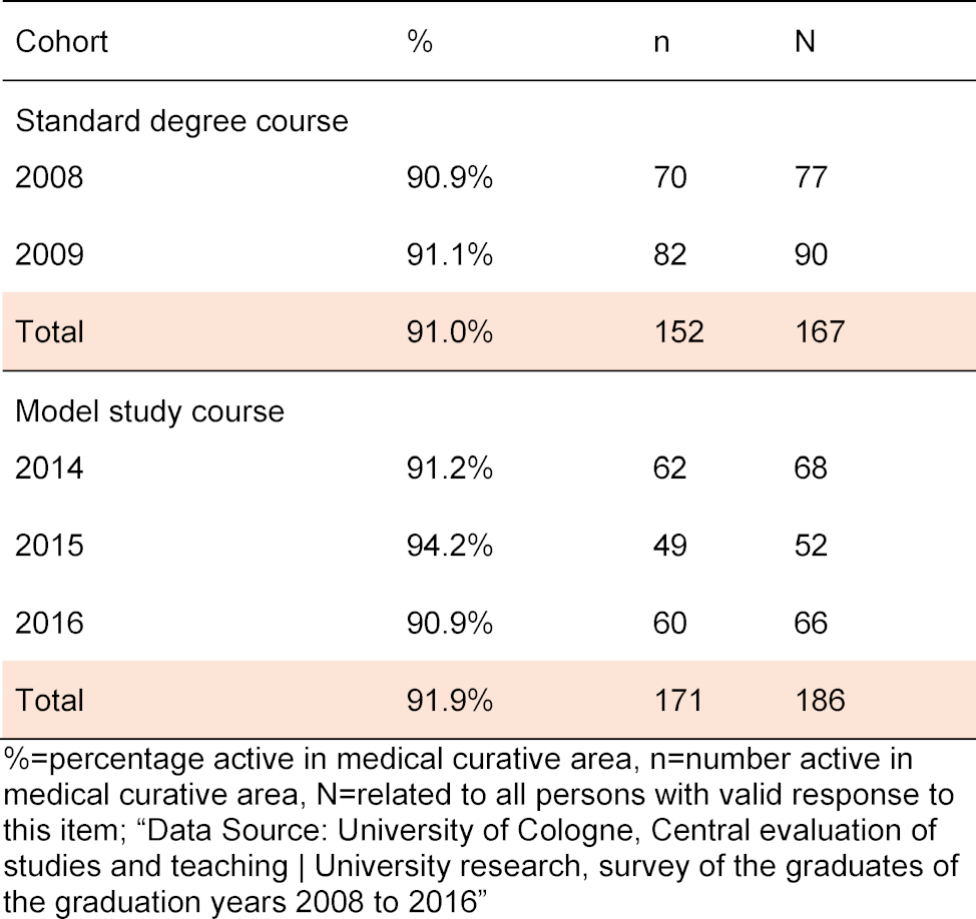
Employment in health care at the time of the survey

**Table 3:**
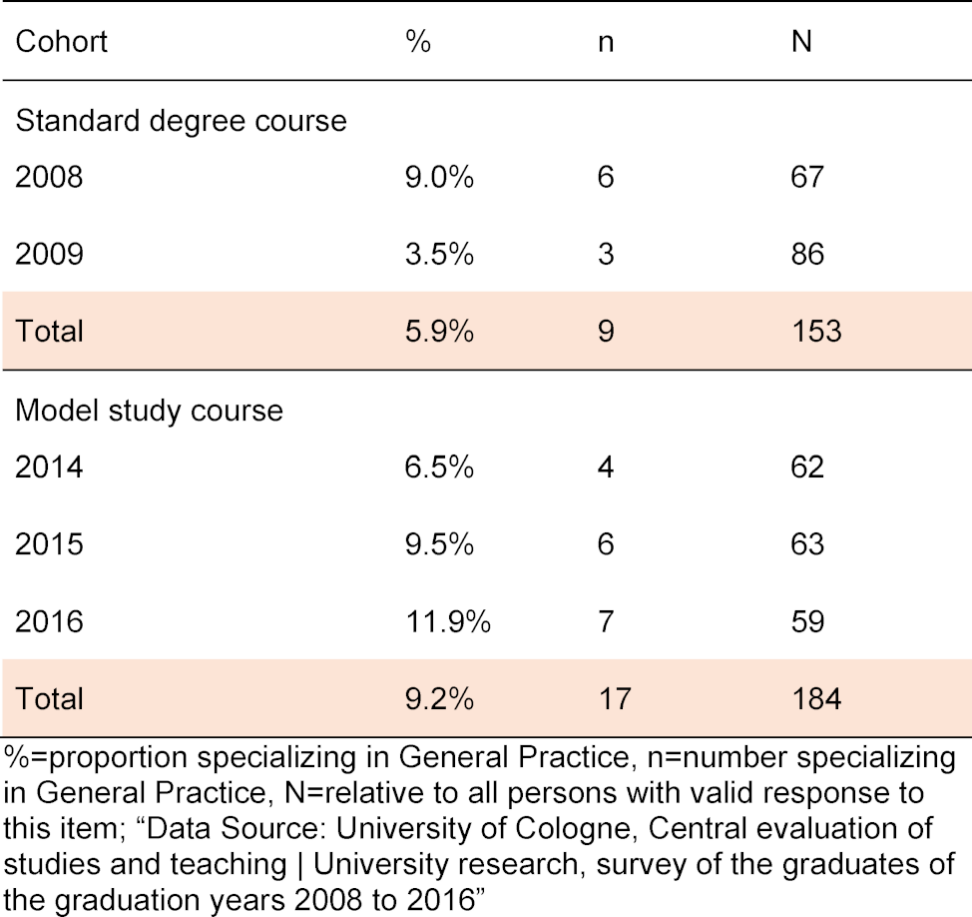
Specialization in General Practice at the time of the survey

**Table 4:**
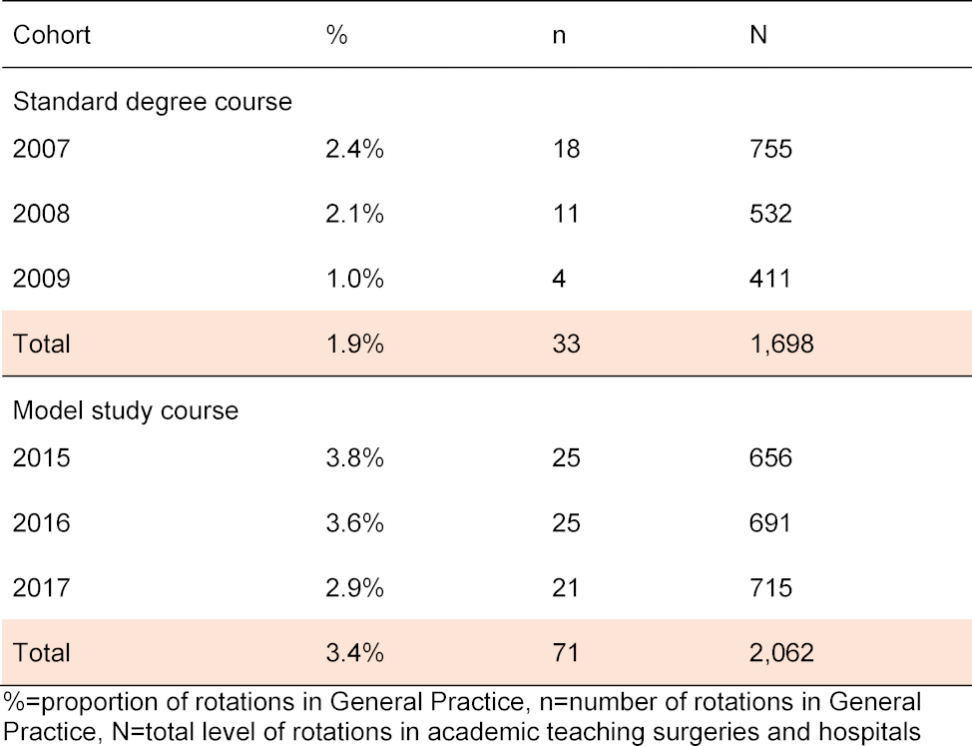
Rotation in General Practice (without rotation at the University Hospital of Cologne)
