# Supplementary material for: Fifteen years of the cologne medical model study course: has the expectation of increasing student interest in general practice specialization been fulfilled?
Source: GMS J Med Educ. 2019 Oct 15;36(5):Doc58. doi: 10.3205/zma001266 (PMC6883249; doi:10.3205/zma001266)
Supplement: HOSPITAL Reform of competence areas in cross-sectional areas [file JME-35-58-s-001.pdf]

HOSPITAL Reform of competence areas in cross-sectional areas

(Recommendation of the Curriculum Commission: Meeting of 11.04.2013)

A cross-sectional area consists of

Cross-sectional block	&	Competence area	&	Competence area	&	Competence area
Q1 Epidemiology, Medical Biometry & Medical IT (5)	&	Clinical studies (9)	&	Heart failure & CHD (9)	&	Allergies (10)
Q2 History, Theory & Ethics of Medicine (5)	&	Ethics in the medical profession (6)	&	Fertility (8)	&	Hereditary diseases (7)
Q3 Health Economics, Health Care System & Public Health (8)	&	Notifiable diseases (5)	&	Looking after diabetes	&	Alcohol & withdrawal (8)
Q4 Infectiology & Immunology (7/10)	&	Transplants & transfusions (6)	&	Rheumatology (10)	&	Fever & sepsis (6)
Q5 Clinical Pathological Conference (7)	&	Renal impairments (9)	&	"The tumor patient" (9)	&	Lymph node enlargement (10)
Q6 Clinical Environmental Medicine (8)	&	Illegal substances & their abuse (5)	&	Dyspnoea (5)	&	Vomiting & diarrhea (10)
Q7 Medicine of Aging & Old People (10)	&	Memory disorder (8)	&	Movement disorders (7)	&	Dizziness (8)
Q8 Emergency Medicine (8/9)	&	Cardiopulmonary resuscitation (7)	&	Polytrauma (6)	&	Upper abdominal complaints (5)
Q9 Clinical Pharmacology & Pharmacotherapy (9)	&	Hypertension Part II (9)	&	Depression (8)	&	Prevention & personalized treatments in oncology (10)
Q10 Prevention & Health Promotion (10)	&	Lifestyle (6)	&	Jaundice	&	Blood count changes (7)
Q11 Imaging Procedures, Radiation Treatment & Radiation Protection (5/9)	&	Thyroid (5)	&	Arterial vascular diseases (7)	&	Ven. thrombosis & pulmonary embolism (6)
Q12 Rehabilitation, Physical Medicine & Naturopathy (6)	&	Joint pain (6)	&	Stroke & neurological rehab (8)	&	Cardiac arrhythmia (8)
Q13 Palliative Medicine (10)	&	Symptom control	&	Therapeutic goal change: Ethical decision making & consultation skills (9)	&	Weight loss & fatigue (9)
Q14 Pain Medicine (9)	&	Adherence & self-medication (7)	&	Tumor pain (6)	&	Headache (8)

( ) Semester
